# PPSDT: A Novel Privacy-Preserving Single Decision Tree Algorithm for Clinical Decision-Support Systems Using IoT Devices

**DOI:** 10.3390/s19010142

**Published:** 2019-01-03

**Authors:** Alia Alabdulkarim, Mznah Al-Rodhaan, Tinghuai Ma, Yuan Tian

**Affiliations:** 1 Information Technology Department, King Saud University, Riyadh 11451, Saudi Arabia; 2 Computer Science Department, King Saud University, Riyadh 11451, Saudi Arabia; rodhaan@ksu.edu.sa; 3 School of Computer Software, Nanjing University of Information Science and Technology, Nanjing 210044, China; thma@nuist.edu.cn; 4 Nanjing Institute of Technology, Nanjing 211167, China; ytian@ksu.edu.sa

**Keywords:** privacy-preserving, CDSS, single decision trees

## Abstract

Medical service providers offer their patients high quality services in return for their trust and satisfaction. The Internet of Things (IoT) in healthcare provides different solutions to enhance the patient-physician experience. Clinical Decision-Support Systems are used to improve the quality of health services by increasing the diagnosis pace and accuracy. Based on data mining techniques and historical medical records, a classification model is built to classify patients’ symptoms. In this paper, we propose a privacy-preserving clinical decision-support system based on our novel privacy-preserving single decision tree algorithm for diagnosing new symptoms without exposing patients’ data to different network attacks. A homomorphic encryption cipher is used to protect users’ data. In addition, the algorithm uses nonces to avoid one party from decrypting other parties’ data since they all will be using the same key pair. Our simulation results have shown that our novel algorithm have outperformed the Naïve Bayes algorithm by 46.46%; in addition to the effects of the key value and size on the run time. Furthermore, our model is validated by proves, which meet the privacy requirements of the hospitals’ datasets, frequency of attribute values, and diagnosed symptoms.

## 1. Introduction

Healthcare centers, such as hospitals and clinics, consider Patients’ Health Records (PHR) as an important asset; they are used to record patients’ medical history, and to refer to during diagnosis. The Internet of Things (IoT) in healthcare is also considered a source of medical data, where different IoT devices can read and monitor patient’s vital signs and symptoms [1]. When diagnosing a patient, a physician starts by checking the PHR, physical examination results, laboratory tests, and when available, the IoT device’s readings. Then, a list of possible diseases will be made based on the patient’s symptoms and signs. Last step is to eliminate one disease at a time from the list in a process called differential diagnosis until one disease category is left [2]. Such complex procedure could be simplified by using Clinical Decision-Support Systems (CDSSs). They are computer systems used by physicians to improve the quality of their diagnosing process [3,4]. Although they are considered an aiding tool for physicians, these systems are evaluated based on their accuracy, and not on how they improve physicians’ performances [5].

Nowadays, PHRs are computerized, which allow health institutions to use them in building a classification model for classifying new symptoms. CDSS uses the classification model to assist physicians in diagnosing patients’ symptoms using data mining techniques or knowledge base [6]. Moreover, multiple hospitals may collaborate together to produce a more accurate model by combining their datasets. However, the privacy of shared data is a serious issue [7,8]; therefore, this will violate patients’ privacy [9,10,11,12]. Privacy preserving data mining is to extract hidden patterns from a dataset without exactly accessing it [13]. The study in Ref. [9] have proposed a Privacy-Preserving Clinical Decision-Support System (PPCDSS) using Naïve Bayes. In their work, they have used a cloud to collect and aggregate encrypted historical medical data to prepare them to be used for training the Naïve Bayes Classifier (NBC). Furthermore, the resulted model is kept private at a third party.

In medical applications, many data mining techniques were used to classify different types of diseases; such as heart diseases [14,15], diabetes [16], and lung cancer [17]. Many studies have shown that decision tree algorithms give higher accuracy results, and some of them were compared to NBC. For example, the study in Ref. [18], even though it did not build a decision tree model, their NBC model was the least accurate. Moreover, the comparison study in Ref. [14] has found that C4.5 was the most accurate model between SVM, KNN, and Neural Networks. Another study in Ref. [16] has also compared various classification techniques, such as J48 and Bayesian Networks. Their experiments have shown that J48 gave the best performance accuracy. Finally, the authors in Ref. [15] have used different data mining techniques to detect heart diseases. The J48 model was of a higher accuracy than the NBC model. Therefore, it can be concluded that in the field of medical applications, decision trees are of better accuracy.

Privacy-preserving decision tree is generated using datasets distributed over multiple participants without disclosing them to each other [19]. They usualyy include the use of encryption, secret sharing schemes or other cryptography algorithms. In this study, we propose a privacy-preserving CDSS using single decision trees and homomorphic encryption to help multiple hospitals to collaborate together, via a cloud, in building the classification model of the CDSS without disclosing patients’ records. Our study is focused on preserving the privacy of the datasets while building the decision tree. The proposed model is based on our novel Privacy-Preserving Single Decision Tree (PPSDT) algorithm. Our simulation results show that the performance of the PPSDT algorithm have exceeded the performance of the NBC algorithm in Ref. [9] by 46.67%. Furthermore, we found that the key size would affect the run time; whereas, the key value would not.

We have proposed in Ref. [12] a privacy-preserving model for a healthcare system. Part of the proposed model addresses the privacy and security challenges faced by clinical decision-support systems. We have designed a model, adapted from Ref. [9], to generate a decision tree privately over multiple parties using homomorphic encryption. Our proposed model had met all of the dataset and patients’ privacy requirements. The contributions of our work are threefold:

1. We propose a model for a Privacy-Preserving Clinical Decision-Support System (PPCDSS) which allows hospitals to privately generate the classification model of the clinical decision-support system and to diagnose new symptoms without sharing their datasets.
2. To ensure patients’ privacy, we propose a novel Privacy-Preserving Single Decision Tree (PPSDT) algorithm to generate decision tree models from distributed datasets without disclosing their actual content using homomorphic encryption.
3. With the help of our method, the results show that our model has improved the classification model generation time by 46.67% compared to the current work in Ref. [9].

The rest of the paper is organized as follows: the formulation of the problem in Section 3. Then, a set of notations and preliminaries in Section 4. Description of the proposed PPSDT algorithm in Section 5; followed by their privacy analysis and performance evaluation in Section 6 and Section 7 respectively. A review of the current literature and related work is in Section 2. Finally, the conclusion in Section 8.

## 2. Related Work

The pioneers in the CDSS field are Ledley and Lusted [2]. They have shown how computers can aid physicians in the complicated diagnosing process. In their work, they have used symbolic logic and probabilities. Setting the foundations of the field, several studies came later proposing different CDSS. The work in Ref. [20] was the first to classify congenital heart diseases using Bayesian classifier; they based their model on the patient’s symptoms, electrocardiograph results, and physical exams. Intensive care unit physicians usually face difficulties related to the diagnosing and treatment of infectious diseases; the study in Ref. [21] proposed a number models addressing these issues one of them using Naïve Bayes. The above studies have focused on improving physician’s performance and productivity. However, the authors in Ref. [9] have addressed a different problem. When CDSS works over a network, patients’ sensitive information is at stake. They have proposed a PPCDSS based on NBC to preserve patients’ privacy while using the CDSS. In their model, a cloud would collect and aggregate patients’ symptoms to securely build an NBC. Another study in Ref. [22], proposed a model using fully homomorphic encryption to build a secure CDSS based on naïve bayes.

Homomorphic encryption is ideal for parties who wish to perform certain calculations on their data using a cloud withou revealing the actual values of the data [23]. The study in Ref. [19] has used homomorphic encryption and digital envelope to construct a collaborative decision tree classification model without leaking participants’ datasets to each other. A semi-trusted commodity server was used in Ref. [24] to train vertically split datasets privately. The work in Ref. [25] expressed their decision trees in form of polynomials; they have proposed privacy preserving decision tree model using fully homomorphic encryption. Lindell et al. [26,27] have used oblivious transfer to design a privacy preserving decision tree model from a distributed dataset. The study in Ref. [28] used Secret Sharing Scheme (SSS) to build a model which allows different parties to build a single decision tree by exchanging the proportions required for calculating the entropy and information gain without revealing the true values. Moreover, the study in Ref. [29], proposed a privacy preserving single layer neural network (SLNN) using secure outsources inner-product protocol (SOIP); their model included the use of a cloud and fog computing.

Privacy preserving data mining studies also proposed solutions for prediction models. The study in Ref. [30] proposed disease prediction model that was stored on a cloud; their CDSS model was based on a single layer perception (SLP) learning algorithm. Given a class value, an SLP decides whether an example belongs to it. They have also used their own novel cryptography algorithm that was based on random matrices. In Ref. [31], Gao et al. proposed a privacy-preserving naïve bayes classifier against substitution-then-compare attack. Their model was based on double blinding techniques, additive homomorphic encryption, and oblivious transfer. Furthermore, the study in Ref. [32] proposed POMP, a disease prediction application based on logistic regression and homomorphic encryption. Their application was designed for patients in rural areas, who can submit their symptoms to cloud to predict their disease. Finally, another study in Ref. [33] proposed an efficient privacy preserving disease prediction model based on kNN algorithm.

## 3. Problem Formulations

### 3.1. System Model

**Figure 1:**
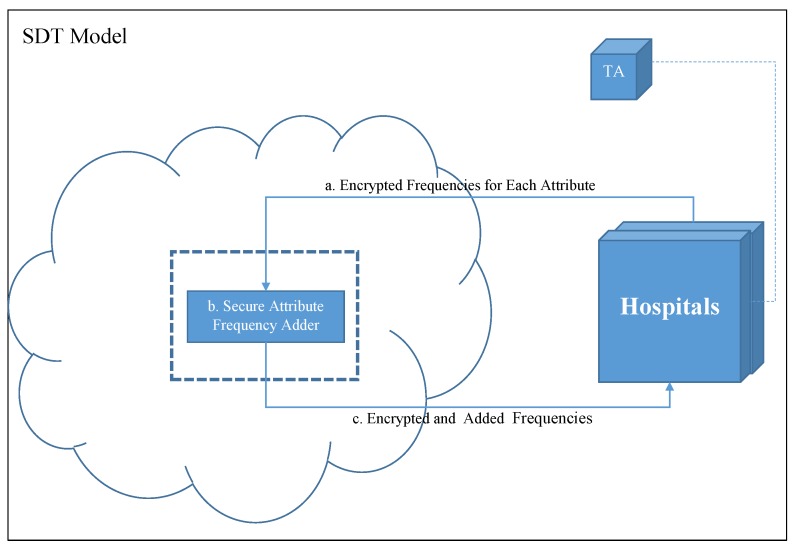
Privacy Preserving Clinical Decision Support System (PPCDSS) model.

In our model, we build a Single Decision Tree (SDT) from the datasets of all participating hospitals’ datasets put together while preserving the patients’ privacy. The main parties in our model are the Hospitals, the Trusted Authority (TA), and the Cloud. Figure 1 shows the layout of the system parties and their relations.

1. Trusted Authority (TA): key distribution and management during setup is handled by the TA.
2. Hospitals: provide the system with the needed proportion of the historical medical data (HMD).
3. Cloud: Securely sums the frequency of attribute values, f, and returns them back to the hospitals.

### 3.2. Attacker Model

An adversary A’s goal is to intercept the communication between the cloud C and the hospital H. A may acquire the encrypted f and nonce. Intercepting the former, A will try to decipher f to obtain the values of the counts. Whereas, with the latter, by deciphering the nonce, A will obtain the nonce value. Furthermore, it is assumed that any hospital H will provide the cloud C with legitimate attribute value frequencies. Finally, we assume no collusion occurs between the system parties that would result in disclosing patients’ personal health records since they are not part of the transmitted data.

### 3.3. Privacy Requirements

The patients’ records and symptoms are considered the main asset which should be kept private. Furthermore, the f values could reveal sensitive information about the hospital’s dataset. Therefore, the following privacy requirements must be met to insure the privacy of hospitals’ patients.

1. **Privacy of the hospitals’ datasets:** each hospital records patients’ medical history and diagnosis in their databases forming a dataset that could be used as a training corpora for the PPSDT algorithm. However, due to the sensitive nature of such records, hospitals will refrain from sharing them, normally. Therefore, their privacy must be assured and preserved in the design of our model.
2. **Privacy of the frequency of attribute values (f):** when each hospital count the required f for calculating the entropy (E) and information gain (IG), they are aware that such data could reveal sensitive information, and normally will not share them willingly unless privacy measures are taken. Therefore, in the design of our model we must satisfy the privacy requirements for keeping them private.
3. **Privacy of the nonce:** due to the fact that in homomorphic encryption the parties use the same public and private key pair, participating hospitals would be considered as semi-honest-but-curious users [34]. Such users are expected to follow the rules, but however, they might be curious to know more. Using the same key pair, would allow any hospital to decrypt another hospital’s data; therefore, each hospital will generate a random nonce to hide the actual values of their data to thwart the threat of having a curious hospital decrypting them. Consequently, the nonce will be encrypted using the cloud’s RSA public key and attached to the sent data.
4. **Privacy of the patients’ diagnosed symptoms:** after diagnosing a patient, a physician would use the CDSS to retrieve the possible diagnosis using patient’s symptoms. However, if no privacy guarantees were offered, then no patient will accept his symptoms to be fed into the system. Therefore, patients’ symptoms must be kept private.

## 4. Notations and Preliminaries

### 4.1. Notation

In this section, we explain the different notations used throughout the paper. Table 1 lists the notations and their meanings.

### 4.2. Preliminaries

In this section, we list the important preliminaries needed to comprehend the construction details of the PPCDSS. We begin with the Paillier cryptosystem for the homomorphic encryption in Section 4.2.1. Then we lay down the C4.5 algorithm for generating decision trees in Section 4.2.2.

#### 4.2.1. Paillier Cryptosystem

Pascal Paillier [35] has designed this homomorphic encryption algorithm. His algorithm is an additive one; thus, can only perform addition operations on ciphertext. The algorithm is composed of the following:

1. Key Generation:
  - Set security parameter *k*
  - Set two large prime numbers *p*, *q*
  - Calculate n=pq
  - Calculate λ=lcm(p−1,q−1)
  - Choose a generator $g\;\in \;Z_{n^{2}}^{*}$
  - Calculate $\mu \;=\;\left(L\left(g^{\mu }mod\;\overset{n^{2}}{N^{2}}\right)\right)\left(-1\right)$
  - Set the public key pk=(n,g)
  - Set the private key sk=(λ,μ)
2. Encryption: given message $m\;\in \;Z_{n}$, and choosing randomly $r\;\in \;Z_{n}^{*}$ evaluate the following:
  $$\begin{array}{cc} E_{PHE}\left(m\right) & =\;g^{m}\cdot r^{n}\;mod\;n^{2} \end{array}$$
3. Decryption: given ciphertext $\;c\;=\;E_{PHE}\left(m\right)$, calculate:
  $$\begin{array}{cc} m & =\frac{L(c^{\lambda }\;mod\;n^{2})}{L(g^{\lambda }\;mod\;n^{2})}\;mod\;n \end{array}$$
4. Addition Property: given two ciphertexts, $\;c_{1}\;=\;E_{PHE}\left(m_{1}\right)$ and $\;c_{2}\;=\;E_{PHE}\left(m_{2}\right)$, which are encrypted with the same key:
  $$\begin{array}{cc} m_{1}+m_{2} & =E_{PHE}\left(m_{1}\right)\;\cdot \;E_{PHE}\left(m_{2}\right)\\ & =g^{m_{1}}\;r_{1}^{n}\;\cdot \;g^{m_{2}}\;r_{2}^{n}\;mod\;n^{2}\\ & =g^{m_{1}+m_{2}}\;r_{1}^{n}\;r_{2}^{n}\;mod\;n^{2} \end{array}$$

#### 4.2.2. Decision Tree Algorithm C4.5

Quinlan’s C4.5 decision tree algorithm is commonly used in classification problems [36]. The main purpose of the algorithm is to construct a decision tree from a dataset of examples and their classes. The algorithm follows the divide and conquer model, where at each step it tries to find the best attribute to split the dataset. This is done by calculating two values: entropy and information gain. To calculate these values, we set $a_{j}$ to be one of the possible values of attribute *A*, where 1≤j≤p; *n* as the number of classes in dataset *D*; and finally a decision class $c_{i}\;\in \;C$, where *C* is the set of decision classes, and 1≤i≤n. See Equations (1) to (3) for more details. In Equation (1), the entropy of the overall dataset is calculated; whereas, in Equation (2), the entropy over a single attribute, *A*, is calculated.

(1) $$\begin{array}{cc} \mathrm{Entropy}(D) & =\sum_{i=1}^{n}\left(\frac{-freq(c_{i},D)}{\left|D\right|}\cdot \log_{2}\frac{freq(c_{i},D)}{\left|D\right|}\right) \end{array}$$

(2) $$\begin{array}{cc} {\mathrm{Entropy}}_{A}\left(D\right) & =\sum_{j=1}^{p}\frac{|a_{j}|}{\left|D\right|}\cdot \mathrm{Entropy}\left(a_{j}\right) \end{array}$$

(3) $$\begin{array}{cc} \mathrm{IG}(A) & =\mathrm{Entropy}\left(D\right)-{\mathrm{Entropy}}_{A}\left(D\right) \end{array}$$

The attribute of maximum information gain will be selected as the best one to split the dataset into *p* partitions. The process will iterate until there are no more partitions.

## 5. The Proposed PPSDT Algorithm

The PPSDT algorithm simply combines the C4.5 algorithm with the Paillier homomorphic encryption to produce a privacy preserving single decision tree algorithm. In this section, we describe the proposed novel PPSDT algorithm by explaining its steps followed by algorithm description.

### 5.1. Design Rationale

Diagnosing patients start by having the physician examining the patient’s vital signs and symptoms and cross-checking it against a list of possible diagnoses. As simple as it seems, such process is time and effort consuming especially with complicated situations [2]. In our model, we propose a design for a PPCDSS model to improve physicians’ productivity. The PPCDSS model is based on our novel Privacy-Preserving Single Decision Tree (PPSDT) algorithm. The latter is a Single Decision Tree (SDT) and Paillier homomorphic encryption algorithms overlapping to generate a decision tree while preserving the privacy of the patients.

SDT is a decision tree that is generated from the datasets of the participating hospitals put together. However, this will suggest to disclose patients’ records to other hospital staff. Therefore, to preserve patients’ privacy, we deploy a cloud to act as a medium in which will securely sum up the proportion values calculated by each dataset owner (hospitals) in order for them to decide at which attribute they should split their data. Tree generation process begins by prompting all hospitals in the system to prepare their counts, f, for each attribute in the dataset. These counts are necessary for calculating the the Entropy (E) and Information Gain (IG). to preserve their privacy, f will be homomorphically encrypted before sending them to the cloud (Figure 1a). At the cloud, the corresponding frequencies received from different hospitals will be added using the Secure Attribute Frequency Adder function (Figure 1b). The securely added f’s will be returned to the hospitals (Figure 1c). At this point, all hospitals have the f over all of the datasets; thus, they can calculate the entropy and information gain.

The process starts by having each hospital count f for each attribute that are needed to calculate the Entropy (E) and Information Gain (IG). Then, the counted f will be homomorphically encrypted and transmitted to the cloud (Figure 1a). There, the frequencies adder (Figure 1b) will securely sum the frequencies of the same attributes; thus, obtain the attribute frequencies of the whole dataset. Then, all securely summed f’s will be propagated back to the hospitals (Figure 1c). Therefore, each hospital will gain the frequencies of the whole dataset; and hence, can calculate the entropy and information gain locally. Finally, the attribute with maximum information gain value will be selected to be the splitting point of their datasets. This process will be repeated until the decision tree is fully generated. As a result, all hospitals will own the same decision tree model, and can use it for diagnosing patients locally.

### 5.2. Construction

The following steps describe the PPSDT algorithm and the role of the cloud and hospitals.

**Figure 2:**
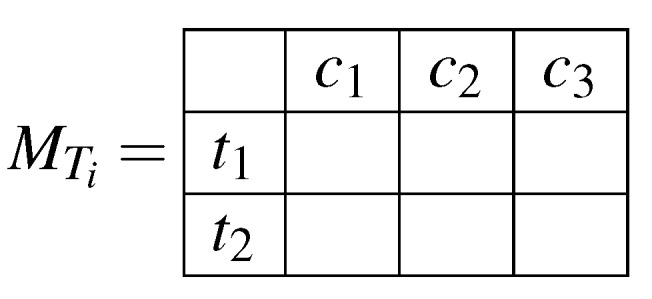
Example matrix for an attribute of two possible values to show their frequencies in respect to three different classes.

1. The cloud will prompt the hospitals to prepare the f for each attribute $T_{i}$. Where f is the frequency of each attribute value per class.
2. The hospitals will prepare the f’s and store them in a matrix ($M_{T_{i}}$). The size of the matrix will be k×l; where *k* is the number of possible values for a given attribute, and *l* is the number of possible decision classes. Each cell $M_{T_{i}}\left[a,b\right]$; where 0≤a<k and 0≤b<l; will hold the frequency of class *b* with attribute value *a* over one hospital’s dataset. This way, we can retrieve the important values for calculating the entropy and information gain from summing different cells together. For instance, the summation of one row will yield the frequency of an attribute value over the dataset, and the summation of all matrix’ cells will yield the dataset size. In Figure 2 we illustrate an example matrix for an attribute of two possible values ($t_{1},t_{2}$), and three different classes ($c_{1},c_{2},c_{3}$).
3. Each hospital will randomly generate a nonce, *z*, and add it to each cell in the above matrix.
4. The nonce will be encrypted using the cloud’s RSA public key.
5. The hospitals will homomorphically encrypt all matrix cells’ values using the public key before sending them to the cloud. The encryption is performed cell by cell because the input of the homomorphic encryption must be an integer or float.
6. The encrypted matrix and nonce are sent to the cloud.
7. The cloud will decrypt the nonce using its own RSA private key.
8. The cloud will subtract the nonce value, *z*, from each cell value.
9. The cloud will homomorphically sum the cells of corresponding matrices then return the results back to the hospitals.
10. Each hospital will homomorphically decrypt the cells of all matrices using the private key.
11. Using the values of the matrix, each hospital will calculate the Entropy and Information Gain (IG), and then pick the attribute of maximum IG as the next node of the tree.
12. The cloud is informed by their selection, then the process will repeat for remaining partitions.

### 5.3. Algorithm Design

The C4.5 algorithm will be adopted to train the decision tree. It should be noted that the training process itself is not distributed, however, the decision tree is trained using a distributed datasets. Algorithm 1 shows how the cloud prompts the hospitals to start the tree building. Because the complete dataset is an aggregation of each hospital’s dataset, each will calculate the required f’s to train the tree, add the nonce, encrypt them homomorphically (using Paillier Homomorphic Encryption), and then send them to the cloud. This process will begin by having each hospital using a 2-dimensional matrix ($M_{T_{i}}$) to store the required f for each attribute $T_{i}$.

In Algorithm 2, we depict the counting of the required f for each attribute $T_{i}$, and storing them in the corresponding matrix. Then, each hospital will homomorphically encrypt each cell value using Paillier Homomorphic Encryption (PHE) after adding the nonce as shown in Equation (4).

(4) $$\begin{array}{c} E_{PHE}\left(M_{T_{i}}\left[a,b\right]+z\right)=\;g^{M_{T_{i}}\left[a,b\right]}\;\cdot \;r^{n}mod\;n^{2} \end{array}$$

Continuing with Algorithm 1, the cloud later will receive all matrices from all hospitals and will homomorphically sum the cells’ values of each corresponding matrices after decrypting and subtracting the nonce. Hence, forming a collection of matrices equal to the number of attributes in the dataset; where each cell will hold a value that represents the frequency of a class with an attribute value over the complete dataset (the datasets of all hospitals put together). Then, all matrices will be returned to the hospitals where each matrix will be decrypted as in Equation (5) for entropy and information gain calculations.

(5) $$\begin{array}{cc} M_{T_{i}}\left[a,b\right] & =D_{PHE}\left(E_{PHE}\left(M_{T_{i}}\left[a,b\right]\right)\right)\\ & =\frac{L({\left[E_{PHE}\left(M_{T_{i}}\left[a,b\right]\right)\right]}^{\lambda }\;mod\;n^{2})}{L(g^{\lambda }\;mod\;n^{2})}\;mod\;n \end{array}$$

To calculate the entropy and information gain, each hospital will extract the required data from the matrices. The calculation of entropy requires the size of the dataset (|D|), and the frequency of each class $c_{i}$ ($freq(c_{i},D)$).

Before calculating the information gain, the entropy of each attribute (Equation (6)) is required. The parameters for the latter equation are the number of matrix $M_{T_{i}}$’s rows (*k*), and the frequency of each attribute value $t_{j}$ ($|t_{j}|$).

(6) $$\begin{array}{cc} {\mathrm{Entropy}}_{T_{i}}\left(D\right) & =\sum_{j=1}^{k}\frac{|t_{j}|}{\left|D\right|}\cdot \mathrm{Entropy}\left(t_{j}\right) \end{array}$$

The results of Equations (1) and (6) will be used to calculate the information gain (IG) (see Equation (7)). The process is repeated for each attribute in the dataset, and the attribute of maximum IG, is selected to be the splitting attribute in the dataset. For each partition, the whole process is repeated until the complete decision tree is generated. These steps are listed in Algorithm 3.

(7) $$\begin{array}{cc} \mathrm{IG}(T_{i}) & =\mathrm{Entropy}\left(D\right)-{\mathrm{Entropy}}_{T_{i}}\left(D\right) \end{array}$$

After the training steps, all hospitals will end up with the same decision tree, where they can use it locally to diagnose new patients. Because of the nature of the decision tree models, only one disease class can be returned for each testing example.

**Algorithm 1:** Build SDT

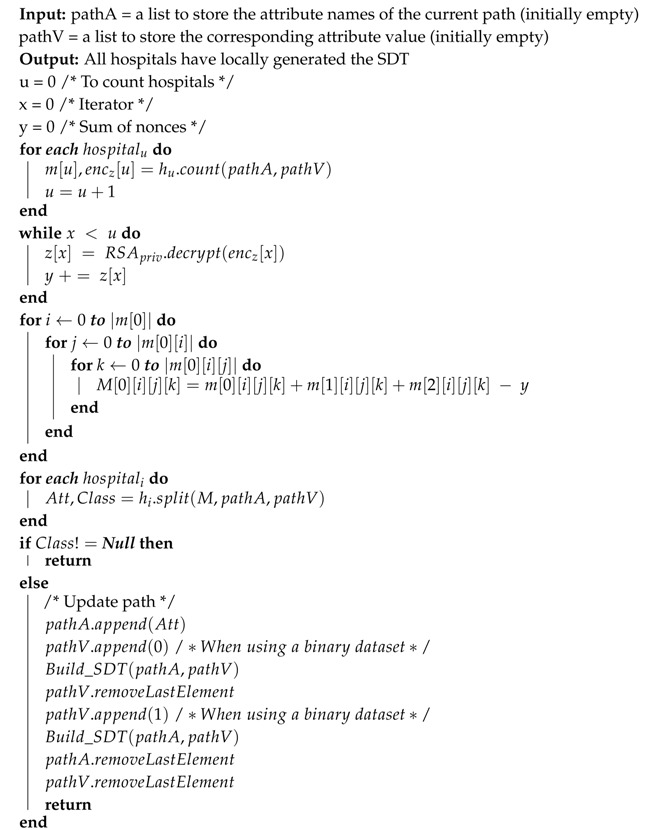




**Algorithm 2:**
Counting
f
and Encrypting the matrix


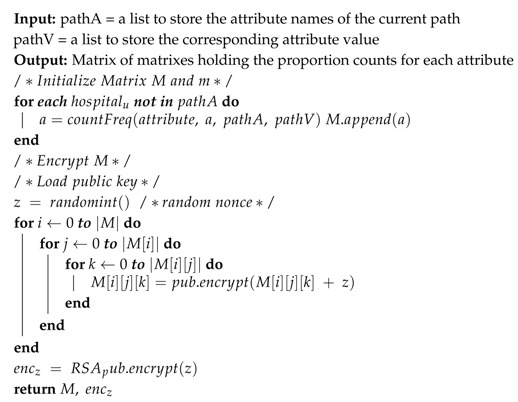




**Algorithm 3:**
Splitting Dataset


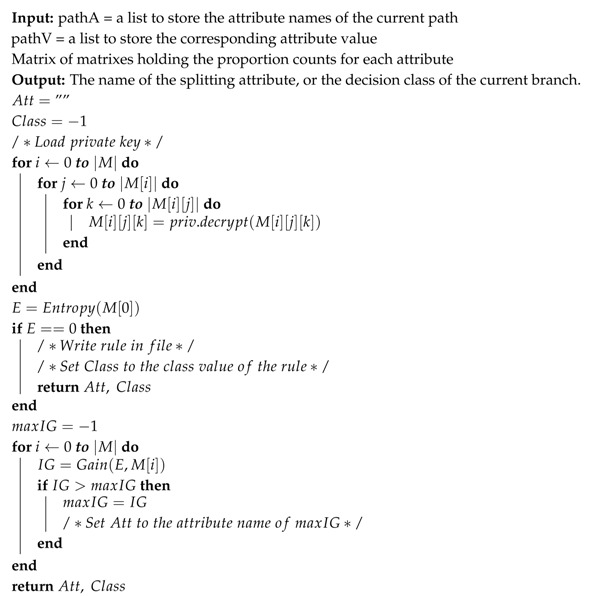



### 5.4. Descriptive Scheme

**Figure 3:**
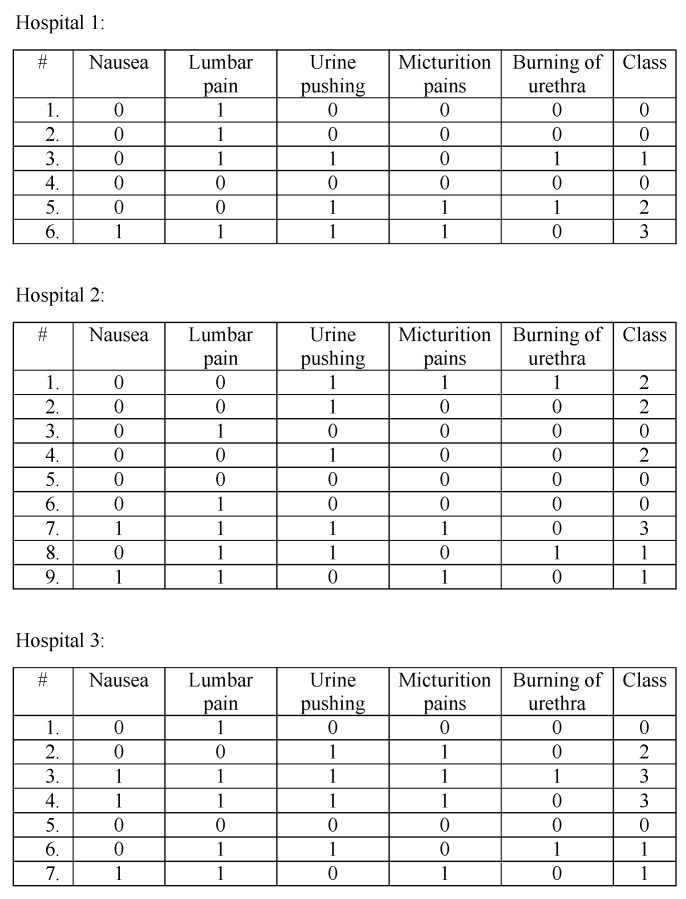
Participating hospitals’ datasets.

In this section, we give a descriptive scheme of the proposed model; where we show how a decision tree is generated through the collaboration of three hospitals. The dataset of the hospitals are in Figure 3 (The dataset was acquired from Ref. [37]; we removed the Temperature attribute because it always gave lowest IG after converting it to 0 = “no fever”, 1 = “fever”). Each dataset has five attributes, and each tuple’s outcome can be one of four classes, 0, 1, 2, and 3 (where 0 = no disease, 1 = Nephritis of renal pelvis origin, 2 = Inflammation of urinary bladder, 3 = Both diseases).

**Figure 4:**
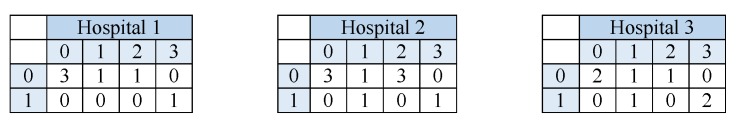
The 2-D matrix for attribute Nausea at each hospital; where it shows the frequency of each attribute value under each class.

To generate a decision tree from the distributed datasets above, we use the assistance of a cloud. First, the required data for entropy and information gain calculations, f, will be prepared at each hospital site. For compactness, the counted f will stored in a two-dimensional matrix for each attribute. The number of columns is decided by the number of classes in the dataset, and the number of rows is decided by the number of possible values for each attribute. One cell value, corresponds to the number of examples in dataset satisfying the column and row values. In Figure 4 we set an example matrix of attribute Nausea for each hospital. In the first matrix (of Hospital 1), cell (0,0) has the value 3; thus, in Hospital 1’s dataset, there are 3 examples that have Nausea = 0 and Class = 0. As a result, the dataset size can be obtained by summing the cell values of one matrix; and the total count of one class value can be obtained by summing the corresponding column. Therefore, the entropy and information gain values can be calculated using these matrices. To preserve the privacy of data held by the matrices, each hospital will homomorphically encrypt the cell values before sending them to the cloud. For simplicity, we omit the homomorphic encryption and nonce from the descriptive scheme.

**Figure 5:**
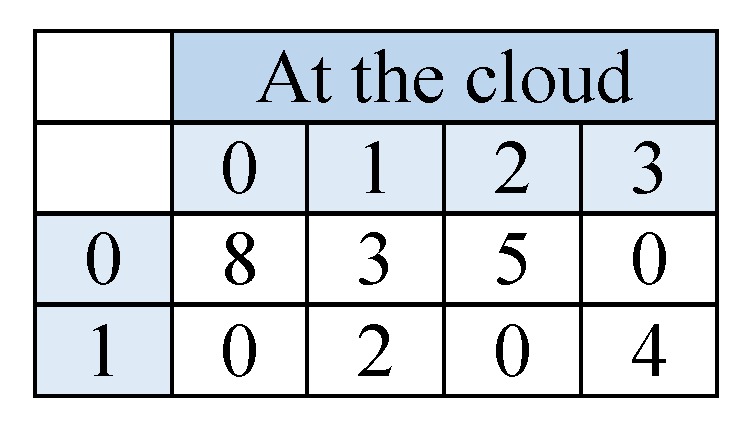
The 2-D matrix for attribute Nausea at the cloud after summing the values of corresponding matrix cells.

At the cloud the matrices of corresponding attributes will be summed in one matrix, see Figure 5. Through homomorphic encryption, the cloud will be able to perform the summation without knowing the actual values of the matrix cells. Afterwards, the cloud will send back the resulted matrices back to the hospitals; there they will be decrypted to retrieve the summation values. Having a matrix for each attribute, each hospital can calculate the values it needs to evaluate the entropy and information gain for the overall datasets.

The below equation is used to calculate the entropy and information gain of attribute Nausea. Equation (1) was used to calculate the entropy value in Equation (8)

(8) $$\begin{array}{cc} \mathrm{Entropy}(D) & =\left(\frac{8}{22}\cdot \log_{2}\frac{8}{22}\right)-\left(\frac{5}{22}\cdot \log_{2}\frac{5)}{22}\right)\\ & -\left(\frac{5}{22}\cdot \log_{2}\frac{5)}{22}\right)-\left(\frac{4}{22}\cdot \log_{2}\frac{4)}{22}\right)=1.949 \end{array}$$

Afterwards, the hospitals will calculate the information gain using Equations (6) and (7).

(9) $$\begin{array}{ll} {\mathrm{Entropy}}_{\mathrm{Nausea}}\left(D\right) & =\frac{16}{22}\cdot [\left(\frac{-8}{16}\cdot \log_{2}\frac{8}{16}\right)-\left(\frac{3}{16}\cdot \log_{2}\frac{3}{16}\right)\\ & -\left(\frac{5}{16}\cdot \log_{2}\frac{5}{16}\right)]+\frac{6}{22}\cdot \left[\left(\frac{-2}{6}\cdot \log_{2}\frac{2}{6}\right)-\left(\frac{4}{6}\cdot \log_{2}\frac{4}{6}\right)\right]=1.3252 \end{array}$$

(10) $$\begin{array}{c} \mathrm{IG}\left(A\right)=\mathrm{Entropy}\left(D\right)-{\mathrm{Entropy}}_{\mathrm{Nausea}}\left(D\right)=1.949-1.3252=0.6238 \end{array}$$

**Figure 6:**
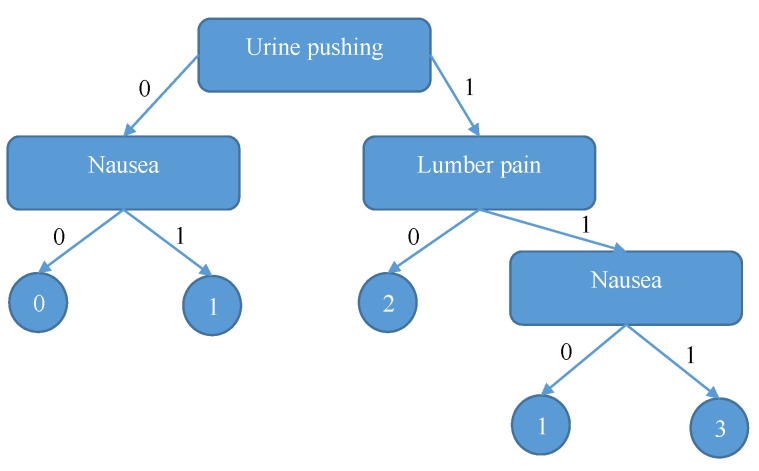
The decision tree generated by collaborating hospitals.

The above steps will be repeated for all attributes in the dataset. As a result, we will find that the attribute with maximum information gain value is “Urine pushing”; therefore, all hospitals will split their datasets there. The process will repeat until the final SDT tree is generated. Figure 6 shows the final decision tree.

Returning to our scenario, we will trace the resulting tree using Bob’s symptoms (Nausea = 0, Lumber pain = 0, Urine pushing = 1, micturition pain = 1, Burning of Urethra = 1) to confirm that Bob’s diagnosis can be inflammation of urinary bladder disease.

## 6. Privacy Analysis

In this section we show how our design goals for preserving the privacy of the patients, hospitals’ datasets and counted frequencies (f) were all satisfied.

### 6.1. Privacy of Hospitals’ Datasets

The primary goal of the PPSDT algorithm, is to generate a single decision tree classification model of all hospitals’ datasets put together without revealing the datasets to each other. Using a cloud to assist the hospitals on calculating the entropy and information gain of the overall dataset has accomplished the goal. Therefore, all hospital’s datasets were protected by not transferring them over the network.

### 6.2. Privacy of Counted Frequencies (f)

The datasets’ f were homomorphically encrypted to hide the sensitive data which could be used to draw important information of the dataset. Hence, the privacy of all datasets and their counted f’s were securely preserved.

(11) $$\begin{array}{c} M_{T_{i}}^{0}\left[a,b\right]=\sum_{k=1}^{n}E_{PHE}\left(M_{T_{i}}^{k}\left[a,b\right]\right) \end{array}$$

Equation (11) shows how the frequencies’ addition, for attribute $T_{i}$ of *n* hospitals, was performed without revealing the actual values of all matrices used to store the frequencies. Where $M_{T_{i}}^{0}$ represents the designated matrix which holds the summation results for attribute $T_{i}$, and $M_{T_{i}}^{k}$ represents the matrix of hospital *k*.

### 6.3. Privacy of the Nonce

The cloud will need to sum the original values of the matrices; therefore, the hospital will encrypt the nonce using the cloud’s RSA public key before sending them to the cloud. There, the cloud will decrypt them, then subtract them from the matrices to restore their original values.

### 6.4. Privacy of the Diagnosed Symptoms

Since the proposed model kept the classification model at the hospitals’ sites, therefore, all diagnosing processes will be performed locally; i.e., the physician will use the CDSS offline. Therefore, the patients’ symptoms will never need to be transferred over the network and be exposed to cyber attacks.

## 7. Performance Evaluation

In this section we discuss the simulation results. All simulations were conducted on a laptop of an intel Core Processor i7 and 8 GB RAM operating on a Linux OS, and Python as a programming language. Our goal is to evaluate the confidentiality and correctness of our PPCDSS through security analysis to insure that the implemented security measures do not affect the accuracy of the data mining techniques used.

**Dataset.** For simulation purposes, a dataset acquired from Ref. [37] will be used for generating the decision trees. For simplification, we have converted the “Temperature” attribute values to 0 = “no fever” and 1 = “fever”. However, our experiments have shown that the “Temperature” attribute always gave the lowest Information Gain (IG) value; and thus, to minimize the computation cost, we have removed it to have a dataset with five attributes: Nausea (N), Lumber pain (LP), Urine pushing (UP), Micturition pains (MP), and Burning of urethra (BU).

**Implementation.** We have used Python as a programming language to simulate the process of generating a single decision tree model over multiple datasets via collaborating hospitals and a cloud. The process starts by the cloud prompting all participating hospitals to start counting the frequencies (f) of all attributes in their datasets. The hospitals will store the f’s in a matrix, then encrypt each element using Paillier homomorphic encryption. All matrices will be returned to the cloud, which in return, will homomorphically add the element values of corresponding matrices. As a result, there will be one matrix for each attribute holding their frequencies over the multiple datasets. Next, the cloud will send back the matrices to the hospitals, and instruct them to calculate the Entropy (E) and Information Gain (IG). At each hospital site, the same steps of decryption and calculation of E and IG will be conducted; and then, all hospitals will select the same attribute to split their datasets, in which it is the one with maximum IG, and add it to the tree. After all hospitals notify the cloud that they have selected an attribute, the cloud will repeat the steps until there are no more partitions. As a result, all hospitals will end up with the same single decision tree.

**Figure 7:**
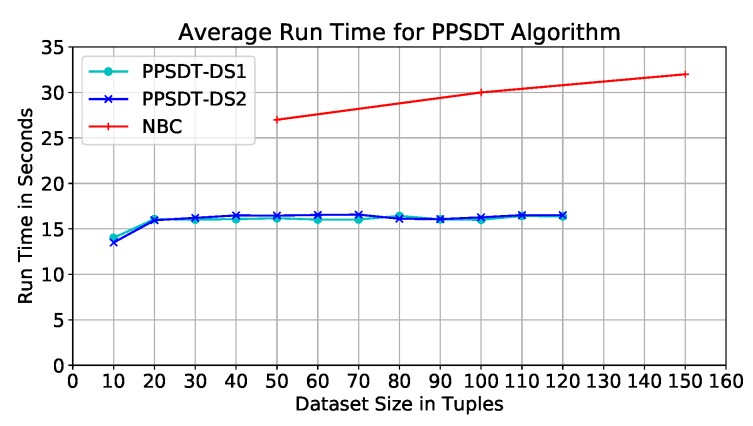
Average run time of the NBC algorithm, and PPSDT algorithm using DS1 and DS2.

**Performance on Dataset.** The code was tested on different dataset sizes; the dataset size represents the size of the overall datasets put together. They varied between 10 and 120 tuples, with an interval of size 10. Our model was tested on two datasets, DS1 and DS2; they are almost 50% similar. Figure 7 depicts the average run time of the model using DS1. We find that the run times of different datset sizes are close varying between 14 and 16 seconds; hence, run time is not affected by the dataset size.

It was found the resulted trees from DS1 for dataset sizes 20–100 tuples are identical, however, the order of branches are different in the sizes 80–100 tuples. This finding explains the peak with dataset size 80 tuples (Figure 7), this shows that the behavior of the tree building algorithm was affected by the values of the dataset itself. Therefore, we test our model on a different dataset, DS2, where we have changed the values of almost 50% of the datasets including dataset size 80 tuples. In Figure 7, we depict the average run time of the PPSDT model with DS2, and we find that the behavior have changed, which proves that the nature of the dataset values affects the behavior of the model. Comparing the two results (Figure 7) we find no major differences in the average run time, and therefore, the datasets’ effects on the output of the model are not major as well.

**Figure 8:**
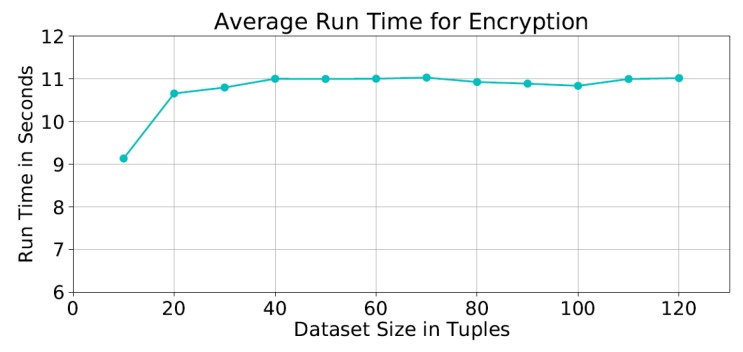
Average run time of the encryption process.

**Performance of Homomorphic Cipher Functions.** The detailed run time shows that the encryption and decryption times forms about 46% of the total run time. It was found that the average encryption time was ≈10 seconds for all dataset sizes (Figure 8).

**Figure 9:**
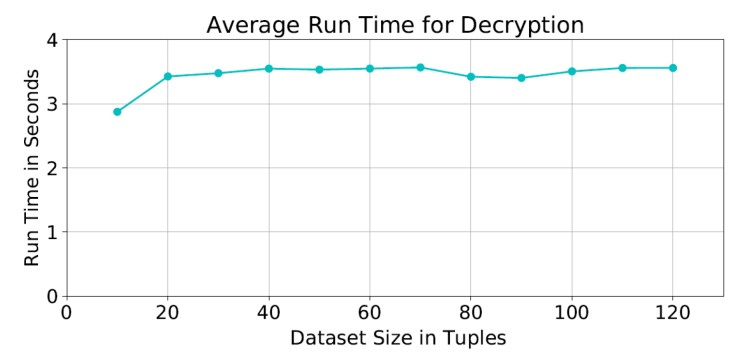
Average run time of the decryption process.

As for the average decryption time, it was ≈3 seconds as shown in Figure 9. We notice that the encryption and decryption times with dataset sizes between 20 and 120 tuples are almost the same. This is due to the fact that the cipher functions are applied to the the same matrix size at each iteration; and therefore not affected by the actual size of the dataset.

**Figure 10:**
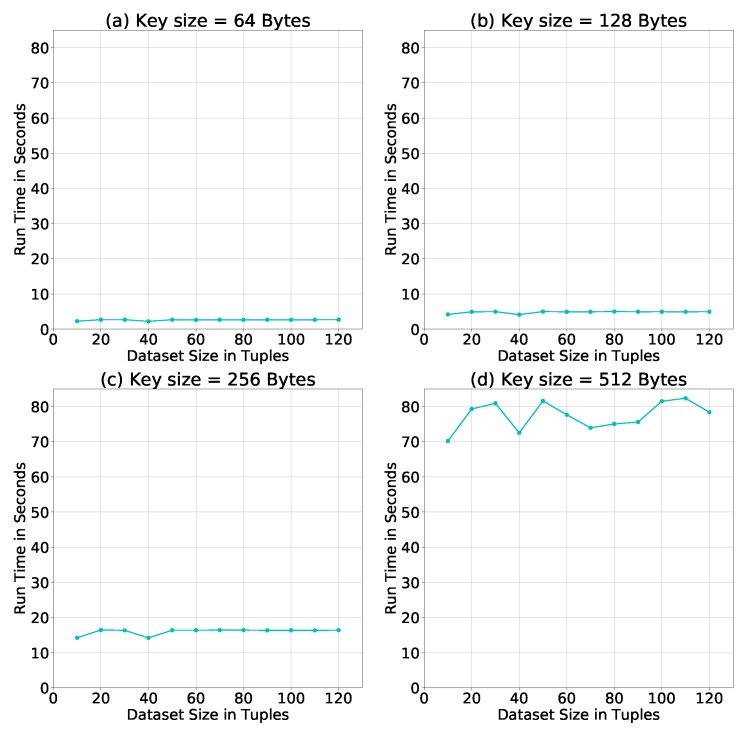
Average run time of the PPSDT algorithm using different key sizes.

**Evaluation of Homomorphic Cipher Keys.** The above simulations were conducted using a key size = 256 Bytes. To test the effect of the key size on the run time of our algorithm, we repeat the simulation using three additional key sizes: 64, 128, and 512 Bytes. Figure 10 shows how the increase in the size of the key increases the run time for each dataset size, and also the difference in run times between dataset sizes. Therefore, it is recommended to choose a key size long enough to be difficult to break, but also not too long to consume system resources and delay results.

**Figure 11:**
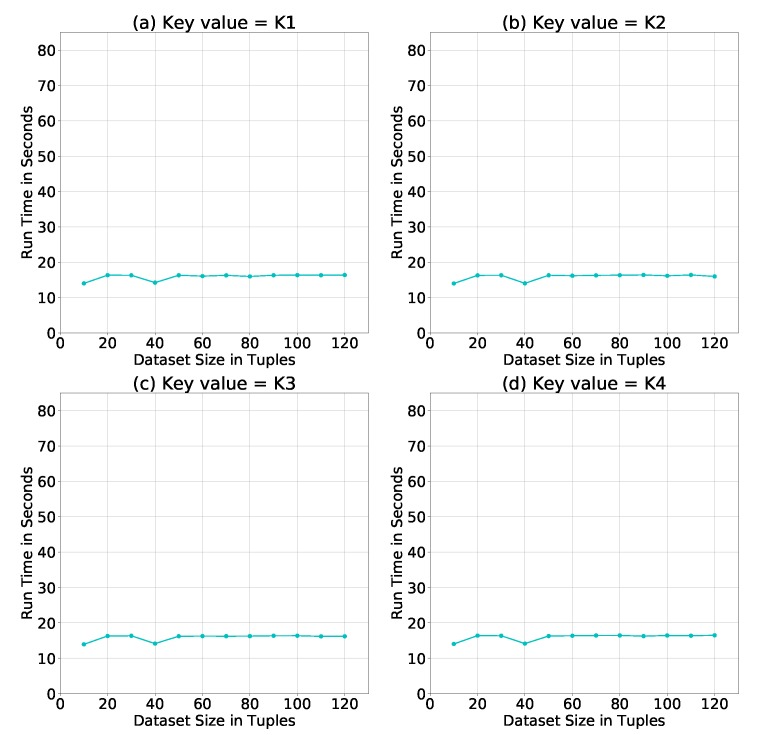
Average run time of the PPSDT algorithm using different key values.

Such comparison leads us to test whether the key value of a certain key size would have an impact on the run time. Figure 11 shows the simulation results of using different key values of size = 256 Bytes. It is clear that the key value had no major impact on the run time of the PPSDT algorithm.

**Evaluation of the resulted SDT model.** To assure that the resulted trees were correct, and were not affected by the encryption and decryption layers, we have used the simple C4.5 algorithm to generate the trees of the overall datasets and of each size, and compared them against the resulted trees of our model; we found that all trees were identical, and thus, our model have produced correct decision trees.

**Performance comparison.** The study in Ref. [9] have used the NBC as a classification algorithm, Paillier and Secure Multiplication protocol [38] for homomorphic encryption, and Additive Homomorphic Proxy Aggregation (AHPA) protocol for secure aggregation. Furthermore, they have used the same dataset we used to test our model with some modifications; as mention earlier, we have removed the “Temprature” attribute whereas they have converted the attribute into 51 attributes, one for each temperature degree. The results of their work have shown that the run time of their model when the dataset size is between 50 and 100 tuples is approximately between 27 and 30 s (Figure 7). Whereas, the PPSDT algorithm run time for the same dataset size range is between 15 and 16 seconds. Thus, our proposed PPSDT algorithm has outperformed the NBC algorithm in Ref. [9] by a 46.67% of improvement. Furthermore, the NBC model reserve the classification model at the third party site; i.e., not the hospitals or the cloud. Thus, for diagnosis, patients’ data will be encrypted and transferred to the third party via the cloud to classify the patients’ symptoms. In our model, we eliminate the risk of having an eavesdropper intercepting the packets holding the patients’ data by distributing the classification model on the hospitals to conduct symptom classification offline.

**Figure 12:**
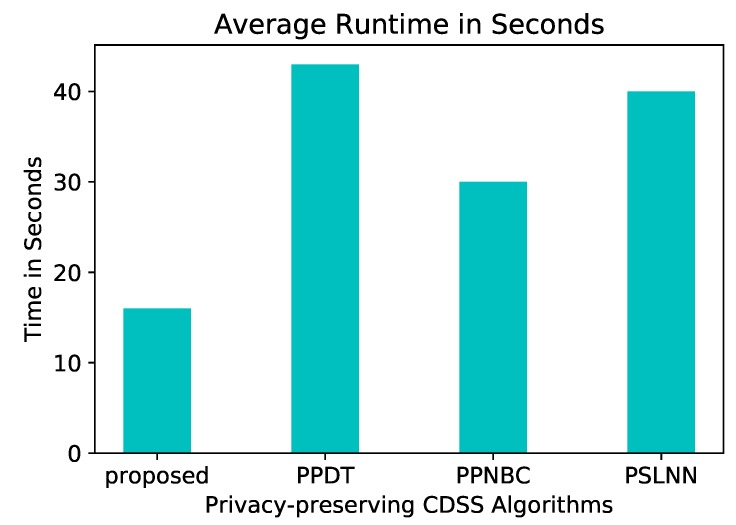
Average runtime compariosn of the PPSDT algorithm.

In a more general comparison, Figure 12 shows the performance of our proposed model next to others in the field: PSLNN [29], PPDT [28], PPNBC [9] in extreme conditions. The idea behind our proposed algorithm and PPDT is similar, the difference between them is that the latter does not use a cloud and considered the parties are arranged in a ring topology, thus, their average runtime is expected to significantly increase as the number of parties increase.

**Scalability.** The increase in the number of participating hospitals in the system will cause an increase in the number of matrices to be summed at the cloud. Furthermore, adding new features to the dataset will add more matrices per hospital. Finally, the change in the number of classes and possible attribute values will affect the size of the matrices. Therefore, the growth in complexity can be calculated as in Equation (12), assuming all attributes have the same number of possible values.

(12) $$\begin{array}{c} C_{PPSDT}=\text{num-of-attributes}\times \text{num-of-classes}\times \text{num-of-attribute-values} \end{array}$$

## 8. Conclusions

Technologies have added efficiency and better quality to healthcare services, hence, caregivers tend to use systems such as CDSS and IoT devices to enhance their performance and patients’ experience. Furthermore, such systems require pateints’ records and symptoms to be transmitted through the network. Such feature imposes a threat on the patients’ privacy for being exposed to different network attacks. In this work, we have proposed a privacy preserving healthcare system, PPCDSS, based on our PPSDT algorithm. Our simulation results have shown that the performance of PPSDT algorithm has outperformed the NBC algorithm in Ref. [9] by 46.67%. Therefore, our model provides a privacy-preserving environment for transmitting patients’ records over the network, and for building a decision tree model without disclosing patients’ information. For future work, we plan to add more security services through Message Authentication Code (MAC), and to generalize our algorithm to accept more forms of datasets.

## Figures and Tables

**Table 1:** Notation.

Symbol	Description
*T*	Set of attributes
$T_{i}$	Single attribute ∈T
$t_{i}$	Possible values for attribute $T_{i}$
$c_{i}$	Possible classes
$M_{T_{i}}$	2-dimensional matrix for storing $T_{i}$’s different f. Rows represent $t_{i}$, and columns represent $c_{i}$
*z*	A unique randomly generated nonce for each hospital
f	Frequency of attribute value
$E_{PHE}$	Paillier encryption function
$D_{PHE}$	Paillier decryption function
*L*	Paillier function
lcm	Least Common Multiplicative
k,μ,g,r,n	Security variables
$Z_{n}^{*}$	A multiplicative group of integers of modulo *n*
$M_{T_{i}}\left[a,b\right]$	$M_{T_{i}}$’s cell value at row *a* and column *b*
*D*	Dataset
|D|	Size of dataset *D*
$freq(c_{i},D)$	The sum of the cell values of all $c_{i}$ columns of all matrices
$|t_{j}|$	The sum of row *j*’s cell values

## Abbreviations

The following abbreviations are used in this manuscript:

CDSS	Clinical Decision-Support System
HMD	Historical Medical Data
IoT	Internet of Things
PHI	Patient Health Information
PHR	Patient Health Record
PPCDSS	Privacy-Preserving CDSS
PPDM	Privacy-Preserving Data Mining
PPRF	Privacy-Preserving SDT
SDT	Single Decision Tree
TA	Trusted Authority
